# Concurrent Validity of a Self-Reported Physical Activity “Vital Sign” Questionnaire With Adult Primary Care Patients

**DOI:** 10.5888/pcd13.150228

**Published:** 2016-02-04

**Authors:** Trever J. Ball, Elizabeth A. Joy, Lisa H. Gren, Janet M. Shaw

**Affiliations:** Author Affiliations: Elizabeth A. Joy, Intermountain Healthcare, Salt Lake City, Utah; Lisa H. Gren, Department of Family and Preventive Medicine, University of Utah, Salt Lake City, Utah; Janet M. Shaw, Department of Exercise and Sport Science, University of Utah, Salt Lake City, Utah.

## Abstract

**Introduction:**

No tool currently used by primary health care providers to assess physical activity has been evaluated for its ability to determine whether or not patients achieve recommended levels of activity. The purpose of this study was to assess concurrent validity of physical activity self-reported to the brief (<30 sec) Physical Activity “Vital Sign” questionnaire (PAVS) compared with responses to the lengthier (3–5 min), validated Modifiable Activity Questionnaire (MAQ).

**Methods:**

Agreement between activity reported to the PAVS and MAQ by primary care patients at 2 clinics in 2014 was assessed by using percentages and κ coefficients. Agreement consisted of meeting or not meeting the 2008 Aerobic Physical Activity Guidelines for Americans (PA Guidelines) of the US Department of Health and Human Services. We compared self-reported usual minutes per week of moderate-to-vigorous physical activity among patients at a primary care clinic in 2014 who reported to PAVS and to MAQ by using Pearson correlation and Bland–Altman plots of agreement.

**Results:**

Among 269 consenting patients who reported physical activity, PAVS results agreed with those of MAQ 89.6% of the time and demonstrated good agreement in identifying patients who did not meet PA Guidelines recommendations (κ = 0.55, ρ = 0.57; *P* < .001). Usual minutes per week of moderate-to-vigorous physical activity reported to PAVS had a high positive correlation with the same reported to MAQ (*r* = 0.71; *P* < .001).

**Conclusion:**

PAVS may be a valid tool for identifying primary care patients who need counseling about physical activity. PAVS should be assessed further for agreement with repeated objective measures of physical activity in the patient population.

## Introduction

Physical inactivity may be the greatest public health problem of the 21st century (1). Assessing and counseling for physical activity in primary health care has led to increased physical activity and improved intermediate health outcomes, such as reductions in lipid levels, blood pressure, fasting blood glucose levels, weight, and incidence of diabetes (2–4). Assessing and counseling about physical activity in primary health care requires that providers and health care systems have a valid tool for assessing patients’ physical activity within clinic workflow (5).

A minimum requirement of a physical activity assessment tool should be to assess whether a patient meets levels of physical activity recommended by the US Department of Health and Human Services’ 2008 Aerobic Physical Activity Guidelines for America*ns* (PA Guidelines) (6–13). PA Guidelines recommends that to promote and maintain health, adults should engage in at least 150 minutes per week of moderate physical activity or 75 minutes per week of vigorous activity, or an equivalent combination of the two (14).

To our knowledge, no tool used in primary health care to assess patient physical activity has been evaluated for concurrent validity in identifying whether or not patients meet recommendations of PA Guidelines (6–9,11). Although not assessed for validity against these recommendations, other brief tools for assessing physical activity among patients show moderate discriminant validity with surveillance of public health physical activity. These tools also show moderate concurrent validity with earlier PA Guidelines recommendations and poor to fair criterion validity with activity measured objectively (6,8,9).

The primary objective of this study was to assess validity of the patient-reported Physical Activity “Vital Sign” questionnaire (PAVS) compared with responses to a concurrently administered Modifiable Activity Questionnaire (MAQ), which has been validated among adults (15,16). A secondary objective was to determine any differences in concurrent validity in patients’ levels of confidence in reporting activity to the PAVS.

## Methods

Participants were volunteer, adult (aged ≥18 y) primary care patients at 2 clinics of Intermountain Healthcare in northern Utah. Patients were ineligible to participate if they did not speak English or if they had diagnosed dementia, because PAVS was administered only in English at the time of this study and because PAVS required cognitively recalling physical activity behavior. Intermountain Healthcare is a nonprofit integrated health care system of 22 hospitals and approximately 160 facilities throughout Utah and southern Idaho. This study was approved by the Intermountain Healthcare Institutional Review Board. We estimated that a sample of 268 participants completing both PAVS and MAQ would provide 95% confidence intervals around a Cohen’s κ for agreement of 46% (7,17).

### Modifiable Activity Questionnaire (MAQ)

MAQ is a modifiable questionnaire, because it queries only activities identified as commonly performed by a study population. We followed steps recently developed by Sternfeld and Goldman-Rosas, a systematic approach, to choose MAQ for evaluating concurrent validity with the PAVS (18). The most notable reason for selecting MAQ to assess concurrent validity with PAVS was that MAQ had the strongest established validity among comparable instruments (19). Although previous studies found that MAQ associated strongly with objectively measured physical activity, MAQ is too lengthy and therefore impractical for use in a primary care clinic setting (7–9).

The MAQ used in this study included moderate-to-vigorous physical activities most commonly performed according to responses to the 2012 Utah Behavioral Risk Factor Surveillance System (20). Intensity of activities was identified as moderate to vigorous according to the Compendium of Physical Activities (21).

### Physical Activity “Vital Sign” (PAVS)

PAVS is a brief (<30 seconds) physical activity questionnaire intended to be administered to every patient at every clinic visit just as vital signs (eg, blood pressure, temperature) and height and weight are measured. PAVS is intended to assess how much light, moderate, or vigorous physical activity a person performs in a typical week. This assessment facilitates determining whether or not a patient meets PA Guidelines recommendations and subsequent patient counseling about physical activity (14).

PAVS asks the following 2 questions, which a patient answers either on a small form when checking in for an appointment or in person to the medical assistant who measures vital signs: 1) “Please describe your level of physical activity, [first by] minutes per day, [followed by] number of days each week,” and 2) “At what intensity (how hard): *light* (like a casual walk), *moderate* (like a brisk walk), or *vigorous* (like a jog/run)?”

Responses are entered in the electronic health record by either medical assistants or the physician. Total minutes per week of activity the patient reports as light, moderate, or vigorous are automatically calculated by the electronic health record by multiplying average minutes per day of physical activity by average number of days per week of activity. Moderate-to-vigorous activity is automatically calculated by summing minutes per week of moderate and vigorous activity.

### Procedures

Patients were recruited by medical assistants after taking patients’ vital signs in private examination rooms. To help control bias of voluntary participation, a standardized invitation to participate was read to patients by medical assistants from an index card. Medical assistants did not mention physical activity while recruiting patients to facilitate attaining a representative sample of participants from the adult clinic population. Patients consented verbally to medical assistants to learn more about the study. Details of the study and MAQ were provided to patients by student research assistants in a private room.

Research assistants first asked patients how confident they felt in reporting their physical activity to PAVS. Confidence was reported on a Likert scale of 1 to 5 (1 = very unsure, 2 = quite unsure, 3 = about 50% sure, 4 = quite sure, and 5 = very sure). After patients reported confidence, research assistants administered MAQ. MAQ was completed an average of 30 minutes after patients reported their activity to PAVS. It was not feasible to randomize the order of administering the PAVS and MAQ assessments because of a lack of privacy in clinic waiting areas and our desire not to interfere with typical patient flow through the clinic.

### Analyses

To help assess how well the volunteer participants in this study represented the total eligible population, we compared demographic characteristics of both groups by using 2-sample tests of proportions.

Correlation and agreement were tested between weekly minutes of physical activity reported to PAVS and MAQ. Correlation and agreement between the 2 instruments were tested only for patients who indicated that activity reported to MAQ was usual for them. This was because PAVS assesses usual activity.

Concurrent validity of PAVS’s ability to identify whether or not patients met PA Guidelines recommendations was assessed by a κ coefficient of binary agreement between the 2 assessment tools’ proportions of patients meeting and not meeting recommended levels. Validity of weekly minutes of activity reported to PAVS was assessed by using Pearson correlation between weekly minutes reported to PAVS and MAQ. Agreement between weekly minutes of activity reported to PAVS and to MAQ was assessed by using Bland–Altman agreement plots (22). Data from outliers (n = 8) were excluded from agreement plots to facilitate visually interpreting agreement plots. These plots had 95% limits of agreement and were unadjusted and adjusted for trend. Correlation and agreement analyses were stratified by patient characteristics.

Participant-reported confidence levels for reporting activity to PAVS were dichotomized into low and high confidence groups according to being either at or below the 50th percentile or at or above the 51st percentile of scores. Differences in PAVS validity by confidence levels was assessed by comparing Pearson correlation coefficients between physical activity reported to PAVS and to MAQ. Statistical differences between correlation coefficients of the low and high confidence groups were tested using a *z* statistic and associated *P* value (23). All analyses were performed with Stata version 11.2 (StataCorp LP). The α level used was .05.

## Results

### Participant characteristics and physical activity behaviors

**Figure 1:**
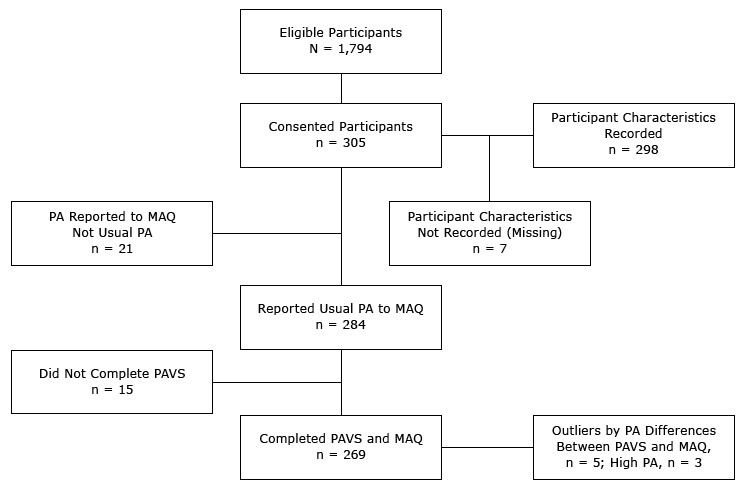
Recruitment and related procedures for selecting participants included for analyses in comparison of the Physical Activity “Vital Sign” questionnaire (PAVS) with the Modifiable Activity Questionnaire (MAQ), Utah, 2104. Abbreviation: PA, physical activity.

A total of 305 patients consented to participate in this study (Figure 1). Demographic characteristics of participants were recorded for 298 participants (Table 1). We found no significant differences in proportions of sex and age groups between volunteer and eligible participants. Most participants were women (61.4%) and white (88.9%); 269 of the 305 participants indicated that physical activity reported to MAQ was usual for them. Patients who reported the greatest weekly minutes of physical activity to both PAVS and MAQ were younger (<55 y) and more educated than patients who reported less physical activity (Table 2).

**Table 1:** Demographic Characteristics of Volunteer Participants^a^ (n = 298) and Eligible Participants^b^ (n = 1,794) in a Study of Concurrent Validity of a Self-Reported Physical Activity “Vital Sign” Questionnaire Among Adult Primary Care Patients, Utah, 2014

Characteristic	Total, n (%)		Male, n (%)			Female, n (%)		
	Volunteer Participants (n = 298)	Eligible Participants (n = 1,794)^c^	Volunteer Participants (n = 115)	Eligible Participants (n = 757)^c^	*P* d	Volunteer Participants (n = 183)	Eligible Participants (n = 1,037)^c^	*P* d
**Total**	298 (100)	1,794 (100)	115 (38.6)	757 (42.2)	.44	183 (61.4)	1,037 (57.8)	.33
**Age, y**								
18–34	33 (11.1)	181 (10.1)	16 (13.9)	69 (9.1)	.52	17 (9.3)	112 (10.8)	.84
35–54	63 (21.1)	391 (21.8)	26 (22.6)	178 (23.5)	.92	37 (20.2)	213 (20.5)	.96
≥55	202 (67.8)	1,222 (68.1)	73 (63.5)	510 (67.4)	.48	129 (70.5)	712 (68.7)	.68
**Educational level^e^**								
Some high school	9 (3.0)	—	4 (3.5)	—	—	5 (2.7)	—	—
High school graduate	59 (19.9)	—	17 (14.9)	—	—	42 (23.0)	—	—
Technical college/other	75 (25.3)	—	28 (24.6)	—	—	47 (25.7)	—	—
University	154 (51.9)	—	65 (57.0)	—	—	89 (48.6)	—	—
**Race/ethnicity^f^**								
Latino/Hispanic	2 (0.7)	—	2 (1.8)	—	—	0 (0.0)	—	—
Asian/Pacific Islander	11 (3.7)	—	5 (4.4)	—	—	6 (3.3)	—	—
Native American	16 (5.4)	—	7 (6.2)	—	—	9 (4.9)	—	—
White	263 (88.9)	—	97 (85.8)	—	—	166 (90.7)	—	—
African American	1 (0.3)	—	0 (0.0)	—	—	1 (0.6)	—	—
No response	3 (1.0)	—	2 (1.8)	—	—	1 (0.6)	—	—

Abbreviation: —, data not available.

a Patients volunteered to participate upon recruitment by medical assistants during a patient’s scheduled doctor appointment. Demographic characteristics of volunteer participants were self-reported.

b Patients were eligible to participate if they spoke English, did not have diagnosed dementia, and had a primary care appointment at the clinics within the timespan of this study. Only characteristics for age and sex were available to report for eligible participants and were extracted from electronic health records.

c Characteristics of eligible participants were acquired from electronic health records, where only age and sex were available.

d *P* values are for 2-sample tests of proportions between characteristics of volunteer participants and characteristics of eligible participants.

e Data missing for one male participant.

f Data missing for 2 male participants.

**Table 2:** Self-Reported Physical Activity Levels Among Participants (N^a^ = 269), by Demographic Characteristics and Assessment Instrument, in a Study of Concurrent Validity of a Self-Reported Physical Activity “Vital Sign” Questionnaire (PAVS) Among Adult Primary Care Patients, Utah, 2014

Characteristic	Reported Usual Min/Wk, Moderate-to-Vigorous Physical Activity^b^			Insufficiently Active^c^			Sufficiently Active^d^		
	PAVS	MAQ	Difference	PAVS, %	MAQ, %	Difference, Percentage Point	PAVS, %	MAQ, %	Difference, Percentage Point
**Total (n = 269)**	150.4	240.8	−90.4	56.9	42.8	14.1	43.1	57.3	−14.2
**Total, outliers excluded (n = 261)^a^**	128.5	214.8	−86.3	57.9	43.7	14.2	42.2	56.3	−14.1
**Confidence in reporting physical activity^e^**									
Low	106.2	209.1	−102.9	70.9	50.6	20.3	29.1	49.4	−20.3
High	170.5	254.2	−83.7	52.0	39.7	12.3	48.0	60.3	−12.3
**Sex**									
Male	214.4	294.7	−80.3	48.0	33.0	15.0	52.0	67.0	−15.0
Female	108.2	203.9	−95.7	63.2	49.7	13.5	36.8	50.3	−13.5
**Age, y**									
18–34	216.4	433.9	−217.5	35.7	21.4	14.3	64.3	78.6	−14.3
35–54	173.9	239.9	−66.0	50.0	42.9	7.1	50.0	57.1	−7.1
≥55	134.0	211.2	−77.2	62.0	46.2	15.8	38.0	53.8	−15.8
**Education**									
Some high school	70.0	238.3	−168.3	77.8	55.6	22.2	22.2	44.4	−22.2
High school graduate	130.0	214.0	−84.0	62.8	56.9	5.9	37.3	43.1	−5.8
Technical college/other	147.9	228.2	−80.3	65.2	46.4	18.8	34.8	53.6	−18.8
University	154.5	240.5	−86.0	50.0	35.5	14.5	50.0	64.5	−14.5

Abbreviation: MAQ, Modifiable Activity Questionnaire.

a Except where indicated, outliers (n = 8) not included in analysis. Outliers were identified when mean differences of reported usual minutes/week of moderate-to-vigorous physical activity exceeded 2.96 standard deviations from the sample’s mean difference in moderate-to-vigorous physical activity (22).

b Defined by the PAVS as “like a brisk walk” (moderate) or “like a jog/run” (vigorous). Defined by the MAQ as activities that were 3 to 6 MET (metabolic equivalent) values according to the Compendium of Physical Activities (moderate) or more than 6 MET values (vigorous) (21).

c Defined as getting less than 150 minutes per week of moderate-to-vigorous activity.

d Defined as getting at least either 150 minutes per week of moderate-to-vigorous activity or 75 minutes per week of vigorous activity.

e Patient-reported confidence in reporting physical activity to PAVS was dichotomized by 50th percentile of confidence scores on a Likert scale of 1 to 5 where 1 = very unsure, 2 = quite unsure, 3 = about 50% sure, 4 = quite sure, and 5 = very sure. A score of 1 to 4 is low and 5 is high.

### Validity and agreement 

PAVS agreed strongly with MAQ 89.6% of the time in identifying patients who were insufficiently active 89.6% of the time (Table 3). PAVS demonstrated moderate agreement for correctly identifying patients as meeting or not meeting PA Guidelines recommendations when accounting for agreement occurring by chance (κ = 0.55, *P* < .001). PAVS correlated strongly with MAQ for assessing patient weekly minutes of activity (*r* = 0.71, *P* < .001).

**Table 3:** Comparison of the Physical Activity “Vital Sign” Questionnaire and the Modifiable Activity Questionnaire by Demographic Characteristics Among Participants (N = 269) in a Study of Concurrent Validity of Self-Reported Physical Activity Questionnaires, Utah, 2014

Characteristic	n^a^	% Agreement, Insufficient Activity	% Agreement, Sufficient Activity	Total, % Agreement	κ (95% CI)	Pearson *r*, Minutes per Week of Moderate-to-Vigorous Physical Activity^b^
**Total**	269	89.6	67.5	77.0	0.55 (0.45–0.64)^b^	0.71
**Total, outliers excluded^c^**	261	90.4	67.4	77.4	0.56 (0.46–0.65)^b^	0.66
**Confidence in reporting physical activity^d^**						
Low	90	88.6	54.3	72.2	0.44 (0.25–0.60)^b^	0.63
High	179	84.6	72.3	79.9	0.60 (0.49–0.71)^b^	0.74
**Sex**						
Male	100	87.9	71.6	77.0	0.53 (0.38–0.69)^b^	0.81
Female	163	90.1	63.4	76.7	0.53 (0.41–0.66)^b^	0.50
**Age, y**						
18–64	149	87.0	67.4	74.5	0.50 (0.37–0.63)^b^	0.75
>64	120	88.9	66.7	80.0	0.60 (0.46–0.74)^b^	0.60
**Education **						
<College degree	129	90.9	61.9	76.7	0.53 (0.39–0.67)^b^	0.69
≥College degree or more	138	87.8	70.8	76.8	0.54 (0.40–0.67)^b^	0.60

Abbreviation: CI, confidence interval.

a The number of participants in each category may not sum to total (n = 269) because information was missing or not reported.

b All are *P* < .001

c Eight participants met predetermined criteria as outliers. Analyses for patient characteristics include data from outliers. Outliers were identified when mean differences of reported usual minutes/week of moderate-to-vigorous physical activity (MVA) exceeded 2.96 standard deviations from the sample’s mean difference in MVA (22).

d Patient-reported confidence in reporting physical activity to the Physical Activity “Vital Sign” questionnaire; dichotomized by 50th percentile of confidence scores on Likert scale of 1–5 where 5 was most confident. Low confidence = score of 1–4; high confidence = 5.

Patients who most frequently self-identified on PAVS as being insufficiently active were patients who reported low confidence reporting physical activity to PAVS (70.9%), patients who were not high school graduates (77.8%), and female patients (63.2%) (Table 2). Agreement for identifying patients as meeting or not meeting PA Guidelines recommendations was strongest for patients with high confidence in reporting activity to PAVS (79.9% of the time; κ = 0.60, *P* < .001) and for older (>64 y) patients (80.0% of the time; κ = 0.60, *P* < .001). Patients who had high confidence in reporting activity to PAVS also had more valid measures of activity (ie, stronger correlation of activity minutes between PAVS and MAQ; *r* = 0.74, *P* < .001). Others that had more valid measures of activity were men (*r* = 0.81, *P* < .001) and younger (18–64 y) patients (*r* = 0.75, *P* < .001) (Table 3).

### Bland-Altman agreement

**Figure 2:**
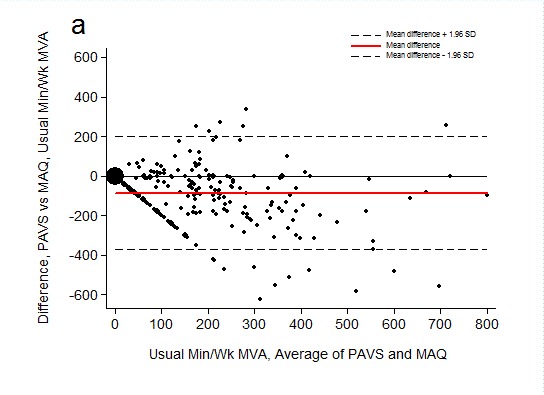

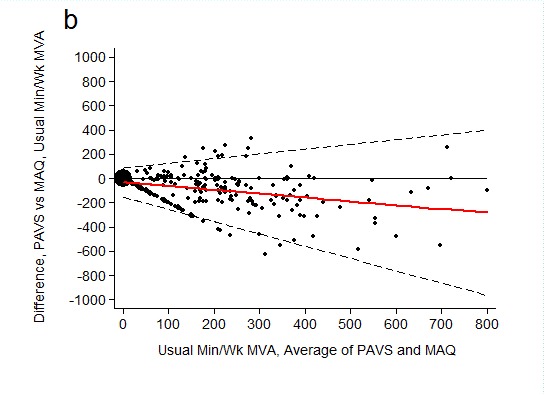
Bland–Altman agreement of usual weekly minutes of moderate-to-vigorous physical activity assessed by the Physical Activity “Vital Sign” questionnaire (PAVS) concurrently with the Modifiable Activity Questionnaire (MAQ). A) Bland–Altman plots with 95% limits of agreement not adjusted for trend. B) Bland–Altman plots with 95% limits of agreement adjusted for trend. Larger plots signify multiple observations with identical coordinates. Abbreviations: MVA, moderate-to-vigorous physical activity; SD, standard deviation.

Bland-Altman agreement between PAVS and MAQ was fair (Figure 2). For total participants, 95% confidence limits were wide (−371.3 to 198.7 minutes/week). Participants reported an average of 86.3 fewer weekly minutes of activity to PAVS (128.5) compared with what they reported to MAQ (214.8; *P* < .001) (Figure 2).

### Participant confidence reporting activity and common activities

Most patients felt “very sure” that their physical activity reported to PAVS was accurate (68%). Weekly minutes of activity assessed by PAVS were more strongly correlated with the same assessed by MAQ among the participants with high confidence self-reporting their activity (*r* = 0.74) compared with low confidence (*r* = 0.63, *P* = .01). The most common activities reported by all participants were walking (66.6%), lifting weights (24.6%), and calisthenics (20.0%) (eg, sit-ups, pushups).

## Discussion

Our study showed that PAVS may be able to identify patients who are insufficiently active according to PA Guidelines recommendations. PAVS accurately identified patients who need counseling for being physically inactive. This was demonstrated by PAVS and MAQ identifying insufficiently active patients 89.6% of the time. An advantage of PAVS over other tools, such as MAQ, is that PAVS takes less than 30 seconds to administer. To our knowledge, this is the first study to evaluate the ability of a patient physical activity assessment tool to compare patients’ physical activity with PA Guidelines recommendations*.*

Primary care providers have an opportunity to improve patient clinical outcomes through counseling for physical inactivity largely because physical inactivity may be the greatest health risk behavior when risk is assessed independent of other health risk behaviors (1). Counseling patients in a health care setting about physical inactivity is facilitated by determining whether patients meet PA Guidelines recommendations, because these guidelines recommendations were designed to prevent disease and promote health (13).

The first step in treating patients for physical inactivity is assessing their activity with a validated instrument. The tool should be feasible for administering in a clinical setting and must be easily understood by patients. Intermountain Healthcare identified the following points as necessary for successfully integrating an assessment of patient physical activity into clinical workflow:

- Educating leaders on leading health risks of physical inactivity.
- Having clinic champions of promoting physical activity (eg, clinic medical directors).
- Making the activity assessment brief while capturing data on activity levels that can be assessed against PA Guidelines recommendations (ie, making the assessment diagnostic as meeting or not meeting recommendations).
- Integrating the assessment into the electronic health record near other vital signs.
- Having patients report their activity on paper when checking in for an office visit. The medical assistant later records the assessment in the electronic health record at the same time other vital signs are recorded.

Other information about integrating a physical activity assessment into regular clinic flow can be found through the Exercise is Medicine initiative of the American College of Sports Medicine (www.exerciseismedicine.org). Once an assessment of physical activity becomes a regular part of clinic workflow, strategies for “treating,” or counseling, for physical inactivity need to be developed. Such strategies may be the provider giving brief counseling or developing a referral network to community physical activity resources provided by Exercise is Medicine.

Valid clinical assessments of physical activity could enable health care providers to treat only patients who do not meet PA Guidelines recommendations. Valid clinical assessment could also minimize measurement error for epidemiologic investigations of physical activity and health outcomes (6,24).

Bland-Altman plots of agreement between physical activity reported to PAVS and to MAQ suggest that dose–response estimates between activity reported to PAVS and clinical health outcomes would be attenuated. This attenuation would occur because PAVS underestimated usual minutes per week of moderate-to-vigorous physical activity by an average of 86.3 minutes per week compared with MAQ. However, although MAQ is strongly correlated with an objective measure of physical activity by accelerometry, MAQ may overestimate activity because it provides more opportunity to report activity than PAVS. That is, MAQ lists more domains, or types, of activity (25,26). The brief PAVS agreed concurrently with the MAQ better than a lengthier physical activity questionnaire agreed concurrently with another instrument (27). In other words, although PAVS is a much shorter physical activity questionnaire than most, it appears to assess moderate-to-vigorous physical activity just as well as lengthier questionnaires.

Physical activity reported by patients in this study agreed with and was correlated most strongly with patients who indicated feeling most confident (ie, upper 50th percentile of confidence rating) reporting their activity. Others have also observed this relationship (28). The accuracy of epidemiologic studies that use physical activity reported by patients and identifiable health outcomes in electronic health records may be improved by understanding factors such as confidence in self-reporting physical activity. Patient confidence in reporting physical activity might improve as patients report more frequently to PAVS, for instance, because PAVS is administered in clinics more regularly. This could be investigated with other studies and may help further assess measurement properties of PAVS.

A strength of this study was our assessing measurement properties of PAVS only in clinics that used this instrument as part of routine patient workflow. PAVS was recommended to assess physical activity in adult patients by administering it to every patient at every visit for approximately 2 years before this study. MAQ, the questionnaire we chose to assess concurrent validity of PAVS, demonstrated the best objective measures of validity among known physical activity questionnaires that measure the same constructs as PAVS (19).

Findings of this study are limited by MAQ’s reliance on self-report. Earlier studies showed that MAQ overestimated physical activity, and it appears to overestimate more than PAVS does (7). A better instrument for assessing concurrent validity of any physical activity self-report assessment might be an instrument with criterion validity assessed by agreement rather than by correlation. As with validation studies of MAQ with criterion measures of physical activity, this study found a strong positive correlation between PAVS and MAQ, but not, for example, by Bland–Altman agreement. The findings of concurrent validity of PAVS in this study are representative only of the patient population that participated.

Finally, in this study, PAVS assessed patient physical activity at only one of 3 levels of intensity: light, moderate, or vigorous. PAVS did not assess activity done at each of these intensities. For this assessment of patient physical activity to be used with, for example, patient electronic health records to examine physical activity–disease relationships, PAVS should assess patient physical activity at all 3 intensities.

Assessing and counseling patients for physical inactivity could significantly improve intermediate health outcomes. This study found strong evidence for the ability of PAVS to correctly identify patients who are insufficiently active according to PA Guidelines recommendations. On average, PAVS underestimated patients’ usual minutes per week of moderate-to-vigorous physical activity compared with MAQ’s assessment. The PAVS should be assessed further for its ability to reliably track trends in patient physical activity. Future study should also assess the validity of using electronic records of patient physical activity for epidemiologic investigations. These assessments would be done most reliably and validly with measures of physical activity that are repeated, objective, and assessed in the patient population.
