# Erratum, Vol 13, February 4 Release

**DOI:** 10.5888/pcd13.150228e

**Published:** 2016-03-03

In the article entitled “Concurrent Validity of a Self-Reported Physical Activity ‘Vital Sign’ Questionnaire With Adult Primary Care Patients,” the second image in Figure 2 was inadvertently omitted because of a technical problem. The figure was corrected on February 9 and appears online at http://www.cdc.gov/pcd/issues/2016/15_0228.htm. We regret any confusion this omission may have caused.

